# Supplementary material for: Clinical predictors of radiological pneumonia: A cross-sectional study from a tertiary hospital in Nepal
Source: PLoS One. 2020 Jul 23;15(7):e0235598. doi: 10.1371/journal.pone.0235598 (PMC7377451; doi:10.1371/journal.pone.0235598)
Supplement: S1 Checklist — (DOC) [file pone.0235598.s001.doc]

STROBE Statement—Checklist of items that should be included in reports of ***cross-sectional studies***

|  | Item No | Recommendation |
| --- | --- | --- |
| **Title and abstract** | 1 | (*a*) Indicate the study’s design with a commonly used term in the title or the abstract  **Study design mentioned**   - **In the title, Page 1, line 2** - **In abstract section: page 2, line 34** |
|  |  | (*b*) Provide in the abstract an informative and balanced summary of what was done and what was found.  **Lines 34-50 (page 2 and 3)**  - **Clinical predictors of radiological pneumonia have been mentioned in the abstract.** |
| Introduction | | |
| Background/rationale | 2 | Explain the scientific background and rationale for the investigation being reported  ***In the introduction section; Line 66-93 (page 3-4***):  **Various studies have been conducted in the pre-vaccine era to define the clinical predictors of radiological pneumonia when the cases associated with radiological pneumonia were usually caused by bacterial agents mostly *Streptococcus pneumoniae* and *Hemophilus influenzae.* The clinical presentation and the radiographic signs of pneumonia may not be the same as found earlier. Considering the change in the epidemiological pattern, the clinical predictors of pediatric pneumonia need reassessment.** |
| Objectives | 3 | State specific objectives, including any pre-specified hypotheses  **Line 90-93 (Page 5):**  **Considering the change in the epidemiological pattern, the clinical predictors of pediatric pneumonia need reassessment. Therefore, this hospital based study was conducted to find the predictors of radiological pneumonia in the post vaccine era.** |
| Methods | | |
| Study design | 4 | Present key elements of study design early in the paper  **Page 5 (line 101)**  **This cross-sectional study was conducted from June-November 2015 in Tribhuwan university Teaching Hospital (TUTH), a tertiary health centre in Kathmandu, Nepal which lies at an altitude of 1400 meters (4600 ft) from the sea level.** |
| Setting | 5 | Describe the setting, locations, and relevant dates, including periods of recruitment, exposure, follow-up, and data collection  **Line 100-112 (page 5 and 6)**  **Setting, location, time of recruitment and cases enrolled have been mentioned.** |
| Participants | 6 | 1. Give the eligibility criteria, and the sources and methods of selection of participants   **Page 5 (line 105-107)**  **Children aged 3-60 months who visited the TUTH at outdoor patient department or emergency unit and presented with fever, cough, and difficulty or fast breathing were enrolled in this study.** |
| Variables | 7 | Clearly define all outcomes, exposures, predictors, potential confounders, and effect modifiers. Give diagnostic criteria, if applicable  **Given in Method section (Pages 5, 6, 7), lines 94-150** |
| Data sources/ measurement | 8* | For each variable of interest, give sources of data and details of methods of assessment (measurement). Describe comparability of assessment methods if there is more than one group  **Given in Method section (Pages 5, 6, 7), lines 94-150** |
| Bias | 9 | Describe any efforts to address potential sources of bias  **Lines 137-138, page 7**  A diagnostic agreement was made between the evaluating pediatrician and radiologist in all cases. |
| Study size | 10 | Explain how the study size was arrived at  **Out of 4211 children visiting the out-patient and emergency unit of pediatric department of TUTH, 1021 patients had fever and were screened for the presence of clinical pneumonia (fever, cough and fast or difficulty breathing) (Result section) and figure 1.** |
| Quantitative variables | 11 | Explain how quantitative variables were handled in the analyses. If applicable, describe which groupings were chosen and why  **All variables were recorded in a excel sheet and mean and percentages were calculated for each variables.** |
| Statistical methods | 12 | 1. Describe all statistical methods, including those used to control for confounding   **Mean and percentages were used. Chi square test was used for categorical variables. Sensitivity, specificity, PPV and NPV were too calculated.** |
|  |  | 1. Describe any methods used to examine subgroups and interactions   **Chi square test was used for categorical variables. Sensitivity, specificity, PPV and NPV were too calculated.** |
|  |  | 1. Explain how missing data were addressed   **There were no missing data** |
|  |  | 1. If applicable, describe analytical methods taking account of sampling strategy   **None** |
|  |  | 1. Describe any sensitivity analyses   **Sensitivity, specificity, PPV and NPV were too calculated.** |
| Results | | |
| Participants | 13* | 1. Report numbers of individuals at each stage of study—eg numbers potentially eligible, examined for eligibility, confirmed eligible, included in the study, completing follow-up, and analysed   **Page 7 (lines 152-155) in Results section:**  **Out of 4211 children visiting the out-patient and emergency unit of pediatric department of TUTH, 1021 patients had fever and were screened for the presence of clinical pneumonia (fever, cough and fast or difficulty breathing). (Given in figure 1)** |
|  |  | 1. Give reasons for non-participation at each stage   **Mentioned in the result section** |
|  |  | 1. Consider use of a flow diagram   **Mentioned in the result section which is clear (flow diagram in figure 1)** |
| Descriptive data | 14* | 1. Give characteristics of study participants (eg demographic, clinical, social) and information on exposures and potential confounders   **Mentioned in the result section** |
|  |  | 1. Indicate number of participants with missing data for each variable of interest   **Mentioned in result section** |
| Outcome data | 15* | Report numbers of outcome events or summary measures  **Lines 161-180, pages 7 and 8** |
| Main results | 16 | 1. Give unadjusted estimates and, if applicable, confounder-adjusted estimates and their precision (eg, 95% confidence interval). Make clear which confounders were adjusted for and why they were included   **See result section, for main results** |
|  |  | (*b*) Report category boundaries when continuous variables were categorized |
|  |  | 1. If relevant, consider translating estimates of relative risk into absolute risk for a meaningful time period   **Not applicable** |
| Other analyses | 17 | Report other analyses done—eg analyses of subgroups and interactions, and sensitivity analyses  **Descriptive analysis comparing radiological pneumonia and non-radiological pneumonia were calculated. Sensitivity, specificity, PPV and NPV were too calculated for each variables of radiological pneumonia.** |
| Discussion | | |
| Key results | 18 | Summarise key results with reference to study objectives  **Pages 9-12, lines 182-253** |
| Limitations | 19 | Discuss limitations of the study, taking into account sources of potential bias or imprecision. Discuss both direction and magnitude of any potential bias  **Pages 11-12, Lines 243-253** |
| Interpretation | 20 | Give a cautious overall interpretation of results considering objectives, limitations, multiplicity of analyses, results from similar studies, and other relevant evidence  **Given in discussion section** |
| Generalisability | 21 | Discuss the generalisability (external validity) of the study results |
| Other information | | |
| Funding | 22 | Give the source of funding and the role of the funders for the present study and, if applicable, for the original study on which the present article is based  **None** |

*Give information separately for exposed and unexposed groups.

**Note:** An Explanation and Elaboration article discusses each checklist item and gives methodological background and published examples of transparent reporting. The STROBE checklist is best used in conjunction with this article (freely available on the Web sites of PLoS Medicine at http://www.plosmedicine.org/, Annals of Internal Medicine at http://www.annals.org/, and Epidemiology at http://www.epidem.com/). Information on the STROBE Initiative is available at www.strobe-statement.org.
