# Supplementary material for: Clinical predictors of radiological pneumonia: A cross-sectional study from a tertiary hospital in Nepal
Source: PLoS One. 2020 Jul 23;15(7):e0235598. doi: 10.1371/journal.pone.0235598 (PMC7377451; doi:10.1371/journal.pone.0235598)
Supplement: Attachment — Submitted filename: Response to Reviewers.docx [file pone.0235598.s002.docx]

To, 13.11.2019

Eric HY Lau, Ph.D.
Academic Editor
PLOS ONE

Dear Sir,

Thank you for providing us with the opportunity to address your valuable comments (Manuscript ID: PONE-D-19-27004). The comments were helpful and we have provided point by point response to the comments and made changes accordingly in the manuscript. We hope this revised version will be acceptable but please let us know if you need some further clarifications.

**Comments:**

1. **Abstract conclusion, tachypnea is not specific at all (Table 2).**

**Action taken:** Thank you for pointing out the mistake. We have corrected this mistake.

2. **The main purpose of the study is to reassess the clinical predictors of radiological pneumonia. In the introduction, please describe the existing known clinical predictors and compare the results in the discussion.**

**Action taken:** We have revised the introduction section as suggested and also have compared the known predictors in the discussion section.

3. **Table 2, please give the % of having the symptoms/signs. For example, the % for ‘noisy breathing’ should be 43/97=44.3%. This will help understanding the estimated sensitivity and specificity.**

**Action taken:** We have given the percentage of sensitivity and specificity in the table as suggested.

4. **Please present the results of the multivariable analysis.**

**Action taken:** The results of multivariate analysis have been given in table 4.

5. **Please explain how combinations of signs/symptoms were determined. Why some combinations were considered (or not considered)?**

**Action taken:** We just wanted to see the difference of sensitivity and specificity of the individual variables and on combining it. We did not find the increase in sensitivity in detecting radiological pneumonia on adding the variables. We also have calculated the combination of other variables which are given below.

| **Combination of variables** | **Sensitivity** | **Specificity** | **PPV** | **NPV** |
| --- | --- | --- | --- | --- |
| Tachypnea + hypoxemia | 67 | 59 | 71 | 54 |
| Tachypnea + auscultatory findings | 20 | 100 | 100 | 45 |
| Hypoxemia+ wheezing | 43 | 50 | 48 | 45 |
| Hypoxemia + bronchial breath sounds | 44 | 100 | 100 | 58 |
| Wheezing+ bronchial breath sounds | 4 | 100 | 100 | 38 |
| Wheezing + hypoxemia + bronchial breath sounds | 7 | 100 | 100 | 58 |

If you suggest, we do not have any problem in omitting this table.

Thanking you,

**Dr. Nagendra Chaudhary**
