# Supplementary material for: Clinical predictors of radiological pneumonia: A cross-sectional study from a tertiary hospital in Nepal
Source: PLoS One. 2020 Jul 23;15(7):e0235598. doi: 10.1371/journal.pone.0235598 (PMC7377451; doi:10.1371/journal.pone.0235598)
Supplement: Attachment — Submitted filename: Response to Reviewers.docx [file pone.0235598.s004.docx]

**REPLY TO THE COMMENTS OF THE REVIEWERS**

**Comments to the Author**

**1. If the authors have adequately addressed your comments raised in a previous round of review and you feel that this manuscript is now acceptable for publication, you may indicate that here to bypass the “Comments to the Author” section, enter your conflict of interest statement in the “Confidential to Editor” section, and submit your "Accept" recommendation.

Reviewer #1: (No Response)

Reviewer #2: All comments have been addressed

Reviewer #3: (No Response**
**2. Is the manuscript technically sound, and do the data support the conclusions?

The manuscript must describe a technically sound piece of scientific research with data that supports the conclusions. Experiments must have been conducted rigorously, with appropriate controls, replication, and sample sizes. The conclusions must be drawn appropriately based on the data presented.

Reviewer #1: Partly

Reviewer #2: Yes

Reviewer #3: Partly**

**Action taken:** We have again amended the manuscript based on the helpful comments from the reviewers. We believe the inferences derived in this revised version are supported by the data and is technically sound.

**3. Has the statistical analysis been performed appropriately and rigorously?

Reviewer #1: I Don't Know

Reviewer #2: Yes

Reviewer #3: No**

**Action taken:** We have again reviewed the statistical section and the numbers. They are correct.
**4. Have the authors made all data underlying the findings in their manuscript fully available?

The** [**PLOS Data policy**](http://www.plosone.org/static/policies.action#sharing) **requires authors to make all data underlying the findings described in their manuscript fully available without restriction, with rare exception (please refer to the Data Availability Statement in the manuscript PDF file). The data should be provided as part of the manuscript or its supporting information, or deposited to a public repository. For example, in addition to summary statistics, the data points behind means, medians and variance measures should be available. If there are restrictions on publicly sharing data—e.g. participant privacy or use of data from a third party—those must be specified.

Reviewer #1: Yes

Reviewer #2: Yes

Reviewer #3: Yes**
**5. Is the manuscript presented in an intelligible fashion and written in Standard English?

PLOS ONE does not copyedit accepted manuscripts, so the language in submitted articles must be clear, correct, and unambiguous. Any typographical or grammatical errors should be corrected at revision, so please note any specific errors here.

Reviewer #1: No

Reviewer #2: Yes

Reviewer #3: (No Response)**

**Action taken:** We have once again checked the language and edited wherever required. Typographical or grammatical errors have been corrected as far as possible by the authors.

6**. Review Comments to the Author

Please use the space provided to explain your answers to the questions above. You may also include additional comments for the author, including concerns about dual publication, research ethics, or publication ethics. (Please upload your review as an attachment if it exceeds 20,000 characters)**

**Reviewer #1: 1) Although most of the reviewer comments were met by the authors in the revision process, I still think that the manuscript does not add any new data into both clinical practice and the literature for further studies.**

**Action taken:** We have mentioned the aim and objective of the study in the manuscript. The diagnosis of pneumonia in children remains an important yet difficult clinical problem. The clinical presentation and the radiographic signs of pneumonia may not be the same as found earlier. Considering the change in the epidemiological pattern, the clinical predictors of pediatric pneumonia need reassessment. Hence, this hospital-based study was conducted to find the predictors of radiological pneumonia. We therefore, feel that this study definitely adds new data to the changing epidemiology of pneumonia.

**2) Since the statistical data analysis has already been explained in Results section, the numbers should not be repeated in Discussion section (all the confidence intervals, percentages and so forth).**

**Action taken:** Thank you for your suggestion. We have removed the repetition and amended the manuscript as advised.

**Reviewer #2: (No Response)**

**Reviewer #3: The calculation of the positive predictive value (PPV) should be done using the Bayes equation, using the prevalence of real pneumonia in the child population in Nepal and not using data from the 2x2 table (see: Linn S. New patient-oriented diagnosis test characteristics analogous to the likelihood ratios conveyed information on trustworthiness. J Clin Epidemiol. 2005; 58 (5): 450–457. doi: 10.1016 / j.jclinepi.2004.07.009 - see equation 2.11).
If this cannot be done by the authors, I suggest not presenting these values in the manuscript.**

**Action taken:** We agree to your suggestion sir. The study was conducted in a tertiary care hospital of Nepal where patients visit from different demographic locations. The data on prevalence of pneumonia in all of the population/ demographical locations of Nepal are scarce. So, we could not apply the Bayes equation to calculate the PPV. We, therefore, as per your suggestion, have removed the PPV and NPV values from the manuscript.
