# Supplementary material for: Clinical predictors of radiological pneumonia: A cross-sectional study from a tertiary hospital in Nepal
Source: PLoS One. 2020 Jul 23;15(7):e0235598. doi: 10.1371/journal.pone.0235598 (PMC7377451; doi:10.1371/journal.pone.0235598)
Supplement: Attachment — Submitted filename: Response to Reviewers Comments.docx [file pone.0235598.s005.docx]

**REPLY TO THE COMMENTS OF THE REVIEWERS**

**Reviewers' comments:

Reviewer's Responses to Questions

Comments to the Author

1. If the authors have adequately addressed your comments raised in a previous round of review and you feel that this manuscript is now acceptable for publication, you may indicate that here to bypass the “Comments to the Author” section, enter your conflict of interest statement in the “Confidential to Editor” section, and submit your "Accept" recommendation.

Reviewer #1: (No Response)

Reviewer #2: All comments have been addressed

Reviewer #3: All comments have been addressed**

**2. Is the manuscript technically sound, and do the data support the conclusions?

The manuscript must describe a technically sound piece of scientific research with data that supports the conclusions. Experiments must have been conducted rigorously, with appropriate controls, replication, and sample sizes. The conclusions must be drawn appropriately based on the data presented.

Reviewer #1: Partly

Reviewer #2: Partly

Reviewer #3: Yes**
**3. Has the statistical analysis been performed appropriately and rigorously?

Reviewer #1: Yes

Reviewer #2: Yes**

**Reviewer #3: Yes
4. Have the authors made all data underlying the findings in their manuscript fully available?

The** [**PLOS Data policy**](http://www.plosone.org/static/policies.action#sharing) **requires authors to make all data underlying the findings described in their manuscript fully available without restriction, with rare exception (please refer to the Data Availability Statement in the manuscript PDF file). The data should be provided as part of the manuscript or its supporting information, or deposited to a public repository. For example, in addition to summary statistics, the data points behind means, medians and variance measures should be available. If there are restrictions on publicly sharing data—e.g. participant privacy or use of data from a third party—those must be specified.

Reviewer #1: Yes

Reviewer #2: Yes

Reviewer #3: Yes
5. Is the manuscript presented in an intelligible fashion and written in Standard English?

PLOS ONE does not copyedit accepted manuscripts, so the language in submitted articles must be clear, correct, and unambiguous. Any typographical or grammatical errors should be corrected at revision, so please note any specific errors here.

Reviewer #1: No

Reviewer #2: Yes

Reviewer #3: Yes**

**Action taken:** We have made some amendments to the earlier submitted version of the manuscript .
**6. Review Comments to the Author

Please use the space provided to explain your answers to the questions above. You may also include additional comments for the author, including concerns about dual publication, research ethics, or publication ethics. (Please upload your review as an attachment if it exceeds 20,000 characters)**.

**Reviewer #1: I still think that the manuscript does not add any new data into the literature and to the clinical practice, either. Diagnosis of pneumonia in children is not an argumentative issue with the defined clinical findings. High respiratory rate, grunting, hypoxemia, decreased breath sounds are already in practice and in literature as signs of pneumonia. Furthermore, the authors did not study on relation of changes in epidemiology and clinical findings. So, they cannot make any comment on that. Epidemiology is the key point in giving decisions on treatment strategies, not in diagnosis of pneumonia, whether it is radiologically approved or not.**

**Action taken:** We acknowledge the reviewer view point on the significance of novelty of this study, however, we differ on its importance of this study in resource scare settings of low-income countries in Nepal where there is requirement of decision making based on the clinical presentations. The diagnosis of pneumonia in children remains an important yet difficult clinical problem especially in low and middle income countries (LMICs) like Nepal. The clinical presentation and the radiographic signs of pneumonia may not be the same as found earlier. Inclusion of vaccination (Hib and PCV vaccines) have changed the epidemiology and presentation of pneumonia in children. Considering the change in the epidemiological pattern, the recent WHO pocket book has also modified the definition of pneumonia. This highlights that the clinical predictors of pediatric pneumonia need reassessment from time to time. Hence, this hospital-based study was conducted to find the predictors of radiological pneumonia in post vaccination era. We therefore, feel that this study highlights on the importance and selection of proper clinical presentation for the diagnosis of pneumonia in low-income countries.

**Reviewer #2: Knowing exactly the symptoms of pneumonia in children is difficult, but it is essential in developing countries.
At the time of respiratory virus pandemic, knowing the local clinical characteristics of presentation of pneumonia becomes even more important.**

**Action taken:** We agree and thank you for highlighting this issue. Although the main objective of our study was also to assess the clinical predictors of radiological pneumonia in children, however, in the current pandemic, it is important to identify both typical and atypical pneumonia cases as early as possible so both preventive and therapeutic measures can be started at an early phase.

**Reviewer #3: Decreased breathing (AOR 3.68; CI 0.99-13.76) was not independently associated, as the lower limit of 95%CI is less than 1. Please correct this, moreover, in all confidence intervals the correct form is 95%CI.**

**Action taken:** Thank you. We have amended the manuscript and inserted “95%” before CI as advised. We understand the adjusted odds ratio of 3.68 with a confidence interval of 0.99 – 13.76 is statistically not significant if you use a very rigid theoretical definition, however, this can be considered as borderline significance. The wide confidence interval is most probably due to low power. We think another study with adequate sample size is required to confirm our findings. We have amended the manuscript accordingly.
