# Supplementary material for: Clinical predictors of radiological pneumonia: A cross-sectional study from a tertiary hospital in Nepal
Source: PLoS One. 2020 Jul 23;15(7):e0235598. doi: 10.1371/journal.pone.0235598 (PMC7377451; doi:10.1371/journal.pone.0235598)
Supplement: Attachment — Submitted filename: Response to Reviewers.docx [file pone.0235598.s006.docx]

**RESPONSE TO REVIEWERS` COMMENTS**
**Reviewers' comments:

Reviewer's Responses to Questions

Comments to the Author

1. If the authors have adequately addressed your comments raised in a previous round of review and you feel that this manuscript is now acceptable for publication, you may indicate that here to bypass the “Comments to the Author” section, enter your conflict of interest statement in the “Confidential to Editor” section, and submit your "Accept" recommendation.

Reviewer #2: All comments have been addressed
Reviewer #3: (No Response)**
**2. Is the manuscript technically sound, and do the data support the conclusions?

The manuscript must describe a technically sound piece of scientific research with data that supports the conclusions. Experiments must have been conducted rigorously, with appropriate controls, replication, and sample sizes. The conclusions must be drawn appropriately based on the data presented.

Reviewer #2: Yes
Reviewer #3: Yes3. Has the statistical analysis been performed appropriately and rigorously?
Reviewer #2: Yes
Reviewer #3: No**

**Action taken:** We have checked the statistical analysis. It is correct.
**4. Have the authors made all data underlying the findings in their manuscript fully available?

The** [**PLOS Data policy**](http://www.plosone.org/static/policies.action#sharing) **requires authors to make all data underlying the findings described in their manuscript fully available without restriction, with rare exception (please refer to the Data Availability Statement in the manuscript PDF file). The data should be provided as part of the manuscript or its supporting information, or deposited to a public repository. For example, in addition to summary statistics, the data points behind means, medians and variance measures should be available. If there are restrictions on publicly sharing data—e.g. participant privacy or use of data from a third party—those must be specified.

Reviewer #2: Yes
Reviewer #3: Yes**

**5. Is the manuscript presented in an intelligible fashion and written in standard English?

PLOS ONE does not copyedit accepted manuscripts, so the language in submitted articles must be clear, correct, and unambiguous. Any typographical or grammatical errors should be corrected at revision, so please note any specific errors here.

Reviewer #2: Yes
Reviewer #3: Yes**
**6. Review Comments to the Author

Please use the space provided to explain your answers to the questions above. You may also include additional comments for the author, including concerns about dual publication, research ethics, or publication ethics. (Please upload your review as an attachment if it exceeds 20,000 characters)

Reviewer #2: (No Response)
Reviewer #3: I repeat, decreased breath sounds (AOR 3.68; 95% CI 0.99-13.76) were not independently associated with radiological pneumonia, please remove it from text! In addition, the result is inaccurate, see the amplitude of the 95%CI.**

**Action taken:** We have removed it from the test as suggested by you and have amended the manuscript.
